# The allogeneic shell technique for alveolar ridge augmentation: a multicenter case series and experiences of more than 300 cases

**DOI:** 10.1186/s40729-022-00446-y

**Published:** 2022-11-01

**Authors:** Peer W. Kämmerer, Jochen Tunkel, Werner Götz, Robert Würdinger, Frank Kloss, Andreas Pabst

**Affiliations:** 1 Department of Oral and Maxillofacial Surgery, University Medical Center Mainz, Augustusplatz 2, 55131 Mainz, Germany; 2 Private Practice for Oral Surgery and Periodontology, Königstraße 19, 32545 Bad Oeynhausen, Germany; 3 Department of Orthodontics, University Hospital Bonn, Welschnonnenstr. 17, 53111 Bonn, Germany; 4 Private Practice for Oral Surgery and Periodontology, Frankfurter Str. 6, 35037 Marburg, Germany; 5 Private Practice for Oral and Maxillofacial Surgery, Kärtnerstraße 62, 9900 Lienz, Austria; 6 Department of Oral and Maxillofacial Surgery, Federal Armed Forces Hospital, Rübenacherstraße 170, 56072 Koblenz, Germany

**Keywords:** Allograft, Cortical bone plate, Alveolar ridge augmentation, Shell technique, Donor site morbidity

## Abstract

**Purpose:**

Allogeneic cortical bone plates (CP) might be used for alveolar ridge augmentation as an alternative to autogenous grafts (AG) and bone substitutes (BS). We report about a multicenter case series and our experiences of more than 300 cases using CP and the shell technique for reconstruction of the alveolar process to illustrate surgical key steps, variations, and complication management.

**Methods:**

Different types of alveolar ridge defects were augmented using the shell technique via CP. The space between the CP and the alveolar bone was filled with either autogenous or allogeneic granules (AUG, ALG) or a mixture of both. Implants were placed after 4–6 months. Microscopic and histological assessments were performed. In addition, space filling using AUG, ALG and bovine BS was discussed.

**Results:**

Scanning electron microscopy demonstrated the compact cortical structure of CP and the porous structure of ALG allowing micro-vessel ingrowth and bone remodeling. Histological assessment demonstrated sufficient bone remodeling and graft resorption after 4–6 months. In total, 372 CP cases and 656 implants were included to data analysis. The mean follow-up period was about 3.5 years. Four implants failed, while all implant failures were caused by peri-implantitis. Next, 30 CP complications were seen, while in 26 CP complications implant placement was possible. CP rehydration, stable positioning by adjusting screws, smoothing of sharp edges, and a tension-free wound closure were identified as relevant success factors. Space filling using ALG and a mixture of AUG/ALG resulted in sufficient bone remodeling, graft resorption and stability of the augmented bone.

**Conclusions:**

CP and the shell technique is appropriate for alveolar ridge augmentation with adequate bone remodeling and low complication rates. Allografts can prevent donor site morbidity and therefore may decrease discomfort for the patient.

## Background

Alveolar ridge atrophy after tooth loss often requires alveolar ridge augmentation before implant placement [1, 2]. In this respect, autogenous bone grafts (AUG) were considered as gold standard for alveolar ridge augmentation since these grafts contain osteogenic cells, collagen, and a multitude of signaling molecules, such as Bone Morphogenic Proteins (BMP) that enable new bone formation without immunological rejection [2–4]. However, definition of a bone graft as gold standard should be dependent on selected criteria, such as augmentation site and volume, remodeling and resorption rate and is controversially discussed [5, 6]. It has been assumed that AUG do not cause further costs such as bone substitutes or allografts. In full-cost accounting, this must be critically discussed since AUG are associated with a potential donor site morbidity leading to indirect health costs such as prolonged treatment or even hospitalization time (e.g., due to iliac crest harvesting) [7, 8]. Further limitations of AUG are an increased resorption rate and a limited availability [8, 9]. Next, surgery time is often prolonged that might be associated with further costs and an increased infection risk [10]. Consequently, the use of allogeneic bone grafts (ALG) from human donors has been established as a suitable alternative to AUG. In general, ALG are considered to have osteoconductive effects while even osteoinduction is discussed and depends on the allograft processing [11–13]. Concerning ALG cleaning and processing, significant differences exist between fresh frozen bone allografts (FFBA) and processed allografts, that can be subdivided into FDBA (freeze-dried bone allograft), MBA (mineralized bone allograft), MPBA (mineralized processed bone allograft), DFDBA (demineralized freeze-dried bone allograft), DBM (demineralized bone matrix) and CBA (cryopreserved bone allograft). This processing results in different clinical outcomes. High complication and failure rates were reported for FFBA [14]. On the contrary, a high osteogenic regeneration potential, natural bone remodeling events, revascularization of regenerated areas, and the absence of foreign body reactions were found in histologically and immunohistochemically evaluations after 5 months of alveolar ridge reconstruction for differently processed ALG [15]. In the last decades, worldwide application of ALG rapidly increased. In Brazil, in 2015, about 20.000 dental patients were treated with ALG [2, 16, 17]. To date, processed allogeneic bone is considered as a safe product [18, 19]. Though, different levels of DNA, cell remnants within the osteocyte lacunae and remnants of the former intra-trabecular fatty tissue were found in different allogeneic bone specimens. It remains unclear whether those DNA and cell remnants have any biological activity. Lorenz et al. reported that these remnants seemed not to influence the recipient in the long term [20]. Neither allergic, immune or human leukocyte antigen (HLA) reactions were documented for processed ALG [21–24]. HLA reactions were exclusively found after FFBA transplantation [17, 25]. Nevertheless, patients must be informed of a theoretical risk of infectious disease transmission (e.g., HCV, HIV) and HLA reactions as part of allograft transplantation. For clinical purposes, ALG is dealing with different application possibilities for alveolar ridge augmentation, such as shell technique. This technique, made popular by Khoury et al., is a widespread surgical procedure that can be performed by autogenous and allogeneic CP in different kinds of alveolar ridge defects [26–30]. There is evidence that allogeneic shell technique seems to be equivalent to autogenous shells concerning vertical and horizontal bone gain [28]. Next to autogenous and allogeneic CP, autogenous dentin blocks, three-dimensionally (3D) printed templates and rigid resorbable barrier systems were reported to be applied as shells with different clinical results [8, 31–34]. Following the principles of guided bone regeneration (GBR), cortical shells are used to create a three-dimensionally secluded and stable space between the shell and the local bone. Additional filling with autogenous or allogeneic granules enables sufficient osseous regeneration [28, 32, 35]. In this context, cortical bone plates have the benefit of long-time stability compared to GBR membranes with its risk of volume reduction caused by soft tissue pressure [36]. Compared to onlay osteoplasty using thick autogenous cortico-spongious grafts, e.g., from the angle of the mandible, shell technique has the advantage that even complex defect geometries can be reconstructed more precisely using two or more bone shells as a further advantage [37]. Next, space filling with granules might induce an improved bone remodeling and vascularization when compared to cortico-spongious grafts [38]. Unfortunately, there is little literature dealing with information about the allogeneic shell technique. We report about a multicenter case series and our experiences of more than 300 cases using CP and the shell technique for alveolar ridge augmentation. The aim of this study was to illustrate surgical key steps, variations, complication management, outcome and surgical experiences in a high case number using allogeneic CP.

## Methods

### Materials

Allogeneic cortical bone plates (CP; maxgraft cortico, diameter 25 × 10 × 1 mm, botiss biomaterials GmbH, Zossen, Germany), allogeneic spongious granules (maxgraft, botiss) and bovine BS (cerabone, botiss) were used. In some cases, relining of the augmented area was performed after implant placement using bovine BS (cerabone) to prevent bone resorption and to gain further bone volume [39]. Next, bovine BS was used in some cases for space filling in a mixture with allogeneic granules and in one case to compare the different techniques of space filling using CP.

### Microscopy and histology

Scanning electron microscopy (SEM) was implemented as reported [35]. Native specimens of CP and allogeneic spongious granules were dehydrated and fixed on specimen trays. After gold-sputtering (SCD 040 sputter-coater; BAL-TEC AG, Leica, Wetzlar, Germany), specimens were visualized by a scanning electron microscope (Philips XL30, Eindhoven, Netherlands).

To assess bone remodeling and graft resorption within the augmentation area, three biopsies were harvested during implant placement by trephine drills in patients with CP shell technique and filling with allogeneic granules or a mixture of autogenous and allogeneic granules. Each biopsy sample was fixed by immersion in 4% buffered formaldehyde (Sörensen buffer) at room temperature (RT) for at least 1 day and subsequently decalcified for about 2 to 3 weeks in 4.1% disodium ethylenediaminetetraacetic acid (EDTA) solution, which was changed every 24 h. After hydration, tissues were dehydrated in an ascending series of ethanol and embedded in paraffin. Serial sagittal sections of 2–3 μm were cut and representative slides were stained with hematoxylin–eosin (HE) and Masson–Goldner trichrome staining (MG). The sections were analyzed using a light microscope (Leica Microsystems GmbH, Wetzlar, Germany).

### Surgery

Alveolar ridge augmentation using CP and the shell technique was performed in patients with Class II–IV defect types as defined by Terheyden [40] under general or local anesthesia. After crestal incision with or without trapezium-shaped or marginal relieving incisions depending on the defect size and geometry, a full-thickness flap was raised. The defect was cleaned from connective tissue residues without decortication of the local bone and the defect size was measured with a sterile caliper. In this context, there is no evidence according decortication benefits [41, 42]. Then, CP were adjusted and trimmed to the correct size by a cutting disc after rehydration in 0.9% saline at room temperature for at least 10 min to increase CP breaking strength and flexibility [35]. After smoothing the edges of the CP via a diamond burr, the screw holes within the CP were drilled outside the oral cavity. Drilling holes in the CP had the same diameter as the adjusting screws. Next, CP were placed as a shell at the alveolar ridge defect and fixed with at least two adjusting screws (diameter 1.0–1.5 mm) to avoid dislocation and rotation. Exemplarily, 1.2 mm osteosynthesis screws (Modus 1.2; Medartis, Basel, Switzerland) can be used. Self-tapping screws should not be used to prevent CP plate fractures. The created space between CP and the alveolar bone was filled with either autogenous, allogeneic spongious granules or a mixture of both. In some cases, space filling was performed by a mixture of allogeneic granules and bovine BS. Autogenous granules were obtained around the augmentation site via bone scraper. Finally, the augmentation area was covered with a collagen membrane of porcine origin. Exemplarily, Jason (botiss biomaterials, Zossen, Germany) or Bio-Gide membrane (Geistlich Biomaterials Vertriebsgesellschaft GmbH, Baden-Baden, Germany) can be used. After periosteal incision, wound closure was stresslessly performed via non-resorbable monofilament threads and single sutures. Reevaluation of the augmented area, screw removal and implant placement were performed 4–6 months later. In some cases, relining of the augmented area was performed immediately after implant placement. Here, the augmented area around the implant was covered with a thin layer of bovine BS and covered with a collagen membrane of porcine origin. This might prevent resorption and further increase the gained bone volume of up to 17% [28, 39].

### Case reports

Four cases are presented to illustrate the surgical key steps, variations, and complication management of CP use. Next, a radiological comparison using cone beam CT scans (CBCT) between autogenous and allogeneic granules, a mixture of both and a mixture of allogeneic granules and bovine BS for space filling was performed.

## Results

### Scanning electron microscopy

**Fig. 1:**
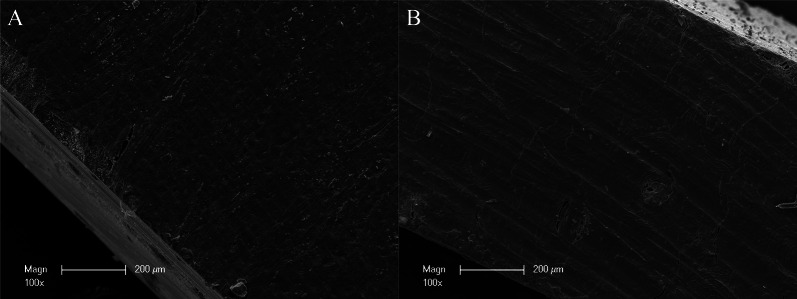
Overview about the microscopic structure and architecture of **A** the surface and **B** the side of an allogeneic cortical bone plate by scanning electron microscopy (SEM) at 100-fold magnification. SEM demonstrating the compact structure of allogeneic cortical bone plate that is suitable for a sufficient barrier and stabilization function

**Fig. 2:**
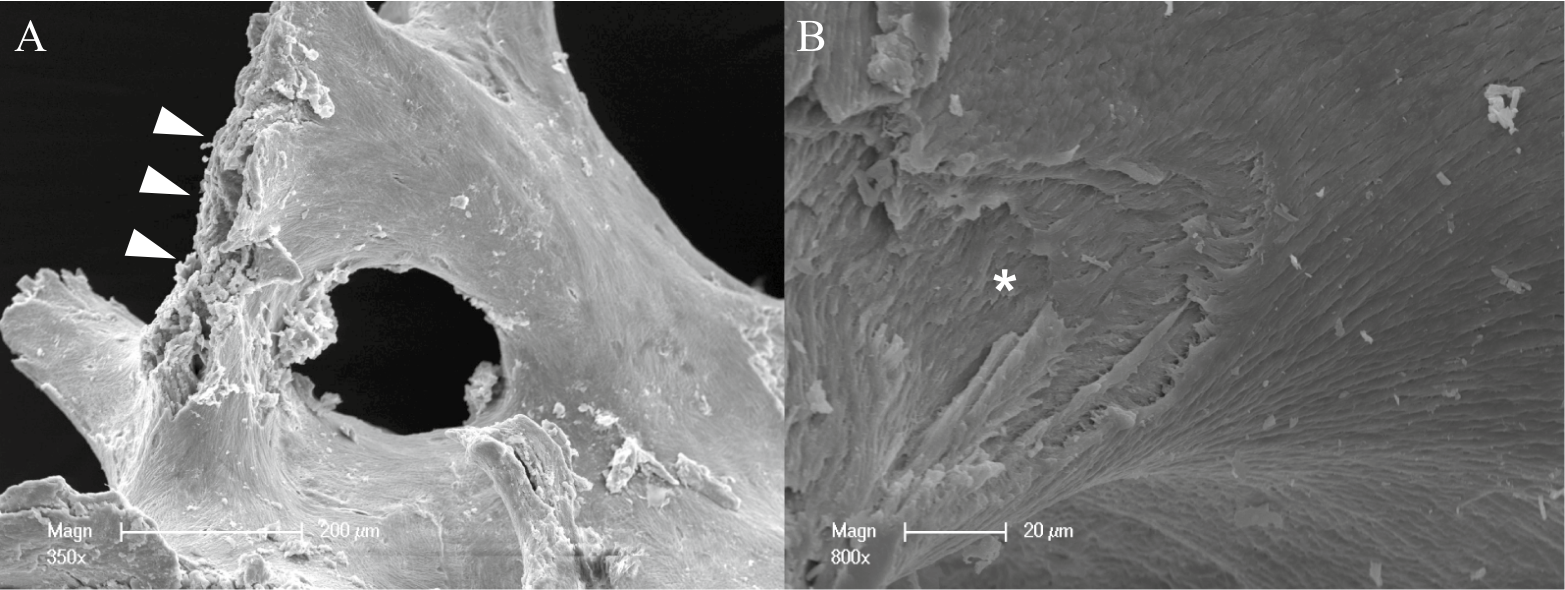
Overview about the microscopic structure and architecture of allogeneic spongious granules by scanning electron microscopy (SEM) in **A** low (350-fold) and **B** high (800-fold) magnification. **A** Allogeneic bone compositions consist of about 5% water and 30% collagen that is visible by the fan-shaped spreading edges (white arrows) and **B** by a multi-layered structure created by collagen fibers (white asterisk). SEM is even highlighting the porous structure of allogeneic spongious granules that is suitable for fast and sufficient micro-vessel ingrowth and bone remodeling

Figure 1 illustrates the microscopic structure of CP by scanning electron microscopy (SEM). In particular, a compact structure of CP is seen that is suitable for a sufficient barrier and stabilization function. Figure 2 illustrates the microscopic structure of allogeneic spongious granules by SEM. These granules consist of about 5% water and 30% collagen that is visible by the fan-shaped spreading edges and by a multi-layered structure created by collagen fibers [43]. SEM is even highlighting the porous structure of allogeneic spongious granules that is suitable for fast and sufficient micro-vessel ingrowth and bone remodeling.

### Surgery

This case series included more than 300 cases using allogeneic cortical bone plates and the shell technique for the reconstruction of horizontal and horizontal/vertical alveolar ridge defects. Different kinds of space filling, specifically autogenous or allogeneic spongious granules or a mixture of both, were used. After 4–6 months, there was a sufficient bone volume gain, remodeling, quality and density in all cases independent from the kind of space filling. CP complication rate was 8.1% (30/372). In detail, types of CP complications were CP dehiscence, early plate and screw exposition, plate fracture, plate loss during implant placement and plate loosening during the healing period with complete CP loss. Implant placement was possible in 26 out of these 30 cases (86.6%). In 4/30 cases with CP complications, specifically with plate loosening during the healing period, complete CP loss (*n* = 3) and dehiscence defect (*n* = 1), implant placement was impossible. To avoid plate loss during implant placement, it might be helpful to leave the adjusting screws during implant placement in place, if this does not interfere with the implant position. In total, more than 650 implants were placed with a follow-up period ranging from 1 to 12 years. The mean follow-up period was about 3.5 years. Here, 4 implants failed cause of peri-implantitis (Table 1).

**Table 1:** Descriptive data concerning case numbers, defect types, the kind of space filling, CP complication rates and kinds of complications, number of inserted implants and implant failures, reasons for implant failure as well as the follow-up periods

Case numbers	372
Defect types	Horizontal, horizontal/vertical
Space filling	autogenous, allogeneic, autogenous/allogeneic mixture, allogeneic/xenogeneic mixture
Time to implant placement	4–6 months
Implant numbers	656
Follow-up period	Ranging from 1 to 12 years
Number of implant failures	4
Reasons for implant failures	Peri-implantitis
Number of CP complications	30
Kind of CP complication	Dehiscence, early plate and screw exposition, plate loss during implant placement, plate loosening
Implant placement possible with CP complications?	Yes, in all cases with exception of 3 cases with plate loosening during the healing period and complete CP loss and 1 case with dehiscence

## Case reports

### Case 1

**Fig. 3:**
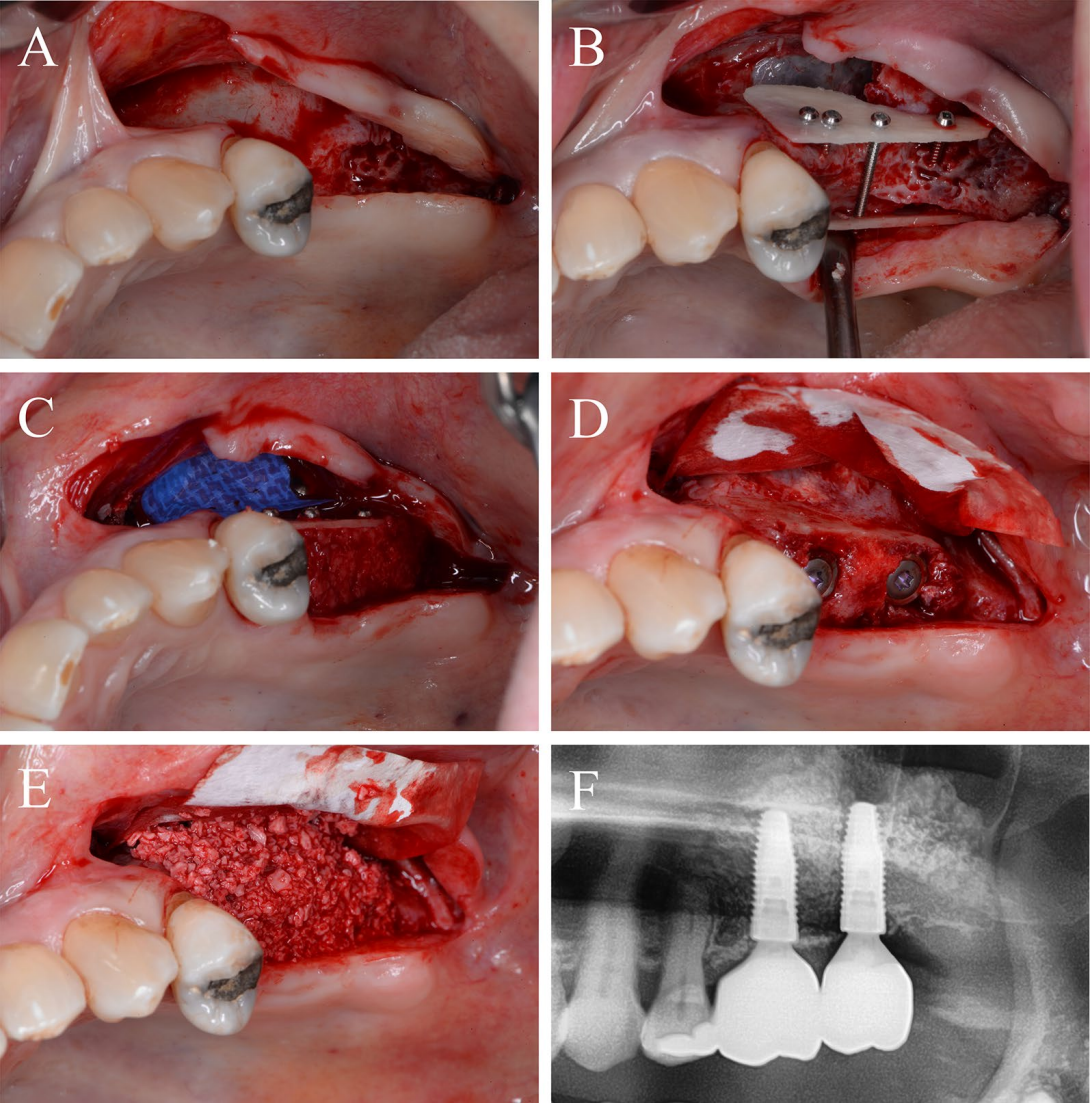
Alveolar ridge augmentation of the posterior maxilla. **A** Initial intraoperative situation with an extended alveolar defect of the posterior alveolar jaw. **B** Intraoperative situation after sinus floor elevation and fixation of two allogeneic cortical bone plates buccal and palatinal using four fixation screws. **C** Intraoperative situation after filling sinus floor with xenogeneic bone substitute, covering it with PTFE matrix and filling the alveolar crest with allogeneic spongious granules. **D** After 4 months, sufficient bone remodeling, screws removed, and implants inserted. **E** Overlining with xenogeneic granules for resorption protection and covered by collagen matrix. **F** X-ray demonstrating inserted implants

Periodontitis and an extended vertical bone defect of about 4 mm and attachment loss resulted in the loss of the first and second molar of the left maxilla in a 60-year-old patient (Fig. 3A). Surgical procedure included sinus floor elevation of the left sinus by preparing a bone lid and sinus filling using bovine BS. CP was divided into two plates and fixed laterally by two screws (diameter 1 mm) on the buccal and palatinal site of the alveolar crest (Fig. 3B). The space between the CP and the alveolar bone was filled by a layered mixture of autogenous and allogeneic granules. Sinus floor window was covered by a PTFA membrane fixed by two pins (Fig. 3C). After 4 months, screws, PTFA membrane and pins were removed; besides, further edges of the CP were smoothed using a rotating instrument. Then, two bone level tapered implants (BLT, 4.8 × 12 mm, Straumann) were inserted in a sufficient bone volume (Fig. 3D). After implant placement, a granular bovine BS was used to cover the augmented area to prevent resorption (relining) and covered by a porcine pericardium membrane (Fig. 3E). Four months after healing, implants were uncovered and gingiva formers were installed along with a roll flap in order to increase the thickness of the keratinized peri-implant mucosa. After one month of healing, the implants were provided with single crowns by the referring general dentist (Fig. 3F). Within the follow-up of 16 months, the case remained stable without complications.

### Case 2

**Fig. 4:**
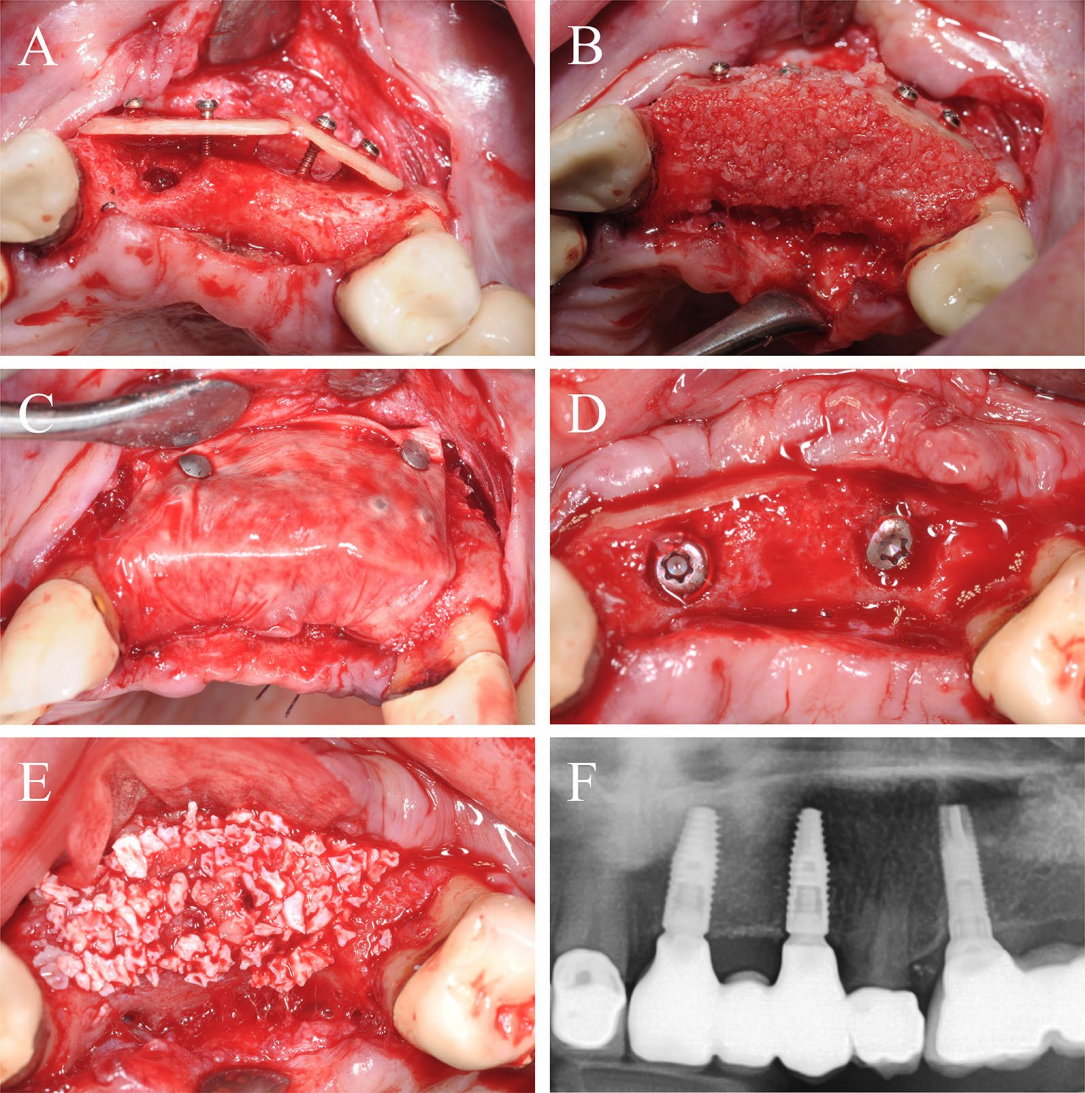
Alveolar ridge augmentation of the anterior maxilla. **A** Initial intraoperative situation with an extended alveolar defect of the anterior alveolar jaw. Two allogeneic cortical bone plates were fixed by fixation screws. **B** Intraoperative situation after filling the defect with allogeneic spongious granules. **C** Covering the augmented area with a porcine collagen membrane. **D** Implant placement 4 months later. **E** Overlining with xenogeneic bone graft to preserve the volume of the augmented area. **F** X-ray demonstrating inserted implants

A 64-year-old patient with multiple missing teeth in the anterior maxilla was referred for implant-borne prosthetic rehabilitation. After clinical and radiological examination, the regions 21–23 showed a severe horizontal bone atrophy preventing implant placement in the intended prosthetic position. A mucoperiosteal flap was raised after crestal incision. The incision line was extended marginally from 13 to 25 with vertical releasing incision distally for tension-free wound closure and to avoid further scars in the esthetic area. First, a CP was split into two fragments. The fragments were attached to the jaw by 1 mm adjusting screws in a curvy line to imitate the new outer contour of the maxilla in regions 21–23 (Fig. 4A). The space between the CP and the native bone was then filled with allogeneic granules (Fig. 4B). The defect site was covered with a barrier membrane and fixed by titanium pins (Fig. 4C). The wound was closed by a combination of horizontal-mattress and single-button sutures. After 4 months, re-entry was performed and the augmented site was uncovered (Fig. 4D). CP were well integrated into the new formed bone tissue and merely visible at re-entry. After recontouring sharp sites of the CP and removing the screws and pins the grafting volume was still well maintained, allowing the insertion of two dental implants (BLT, 4.1 × 12 mm, Straumann) in the correct position according to the treatment plan. Afterwards, bovine BS was used for relining of the grafted area to prevent resorption of the new formed crest and increase the volume stability in the long-term (Fig. 4E). The grafted site was again covered with a pericardium collagen membrane. Following another 4 months, the implants were uncovered, and healing abutments were installed along with a modified apically repositioned flap in combination with roll-flaps and gingival tuber transplantation in region 22 to increase the thickness of attached keratinized gingiva. After another 2 months of healing, the final dental bridge was installed by the referring general dentist (Fig. 4F). Within the follow-up of 25 months, the case remained stable without complications.

### Case 3

**Fig. 5:**
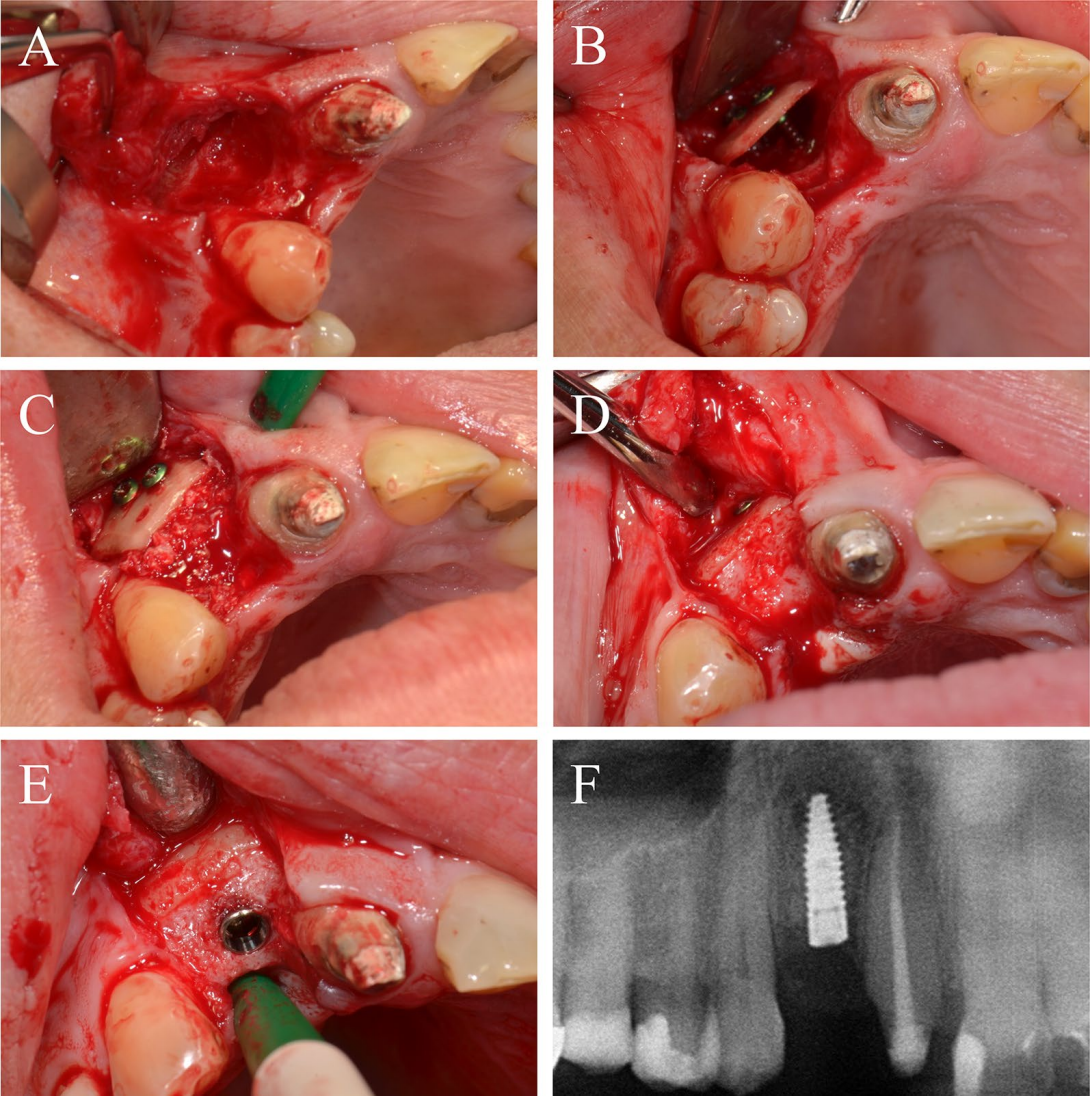
Alveolar ridge augmentation of the anterior maxilla. **A** Initial intraoperative situation demonstrating an extended tub-shaped alveolar defect after a failed augmentation attempt using an autogenous graft from the mandibular angle and postoperative hypesthesia of the mandibular nerve. **B** An allogeneic cortical bone plate was placed and fixed by adjusting screws. **C** The occurred space was filled with allogeneic spongious granules. **D** Four months later, a sufficient bone bed was found and **E** a bone level tapered implant was inserted. **F** X-ray demonstrating the inserted implant

A 43-year-old patient presented herself after single tooth loss of the right maxilla and an extended tub-shaped alveolar ridge defect wishing implant-based prosthetic restoration. The initial treatment concept included bone augmentation with an autogenous bone graft harvested from the oblique line of the angle of the mandible. After graft harvesting, patient suffered from recurrent hypoesthesia of the alveolar nerve for nearly 2 months. Since the autogenous bone graft was lost, a second bone augmentation was performed using CP and allogeneic granules in accordance with patient’s wishes. After palatinal-shifted crestal incision, a full mucoperiosteal flap was raised and the failed graft was removed (Fig. 5A). The CP was prepared outside of the oral cavity and installed over the defect using two adjusting fixation screws (each 1.5 × 8 mm; Fig. 5B). The defect was filled with allogeneic granules (Fig. 5C) and covered with a collagen membrane and two PRF-matrices. Four months later, a sufficient bone regeneration with integration of the CP was seen. In accordance, screws were removed and a dental implant (BLT, 3.3 × 10 mm, Straumann) was installed (Fig. 5D–F). After 4 months of healing, a gingiva former was installed and—after 2 weeks of further healing—a single crown was installed. With a follow-up of about 4 years, this case remained stable without complications.

### Case 4

**Fig. 6:**
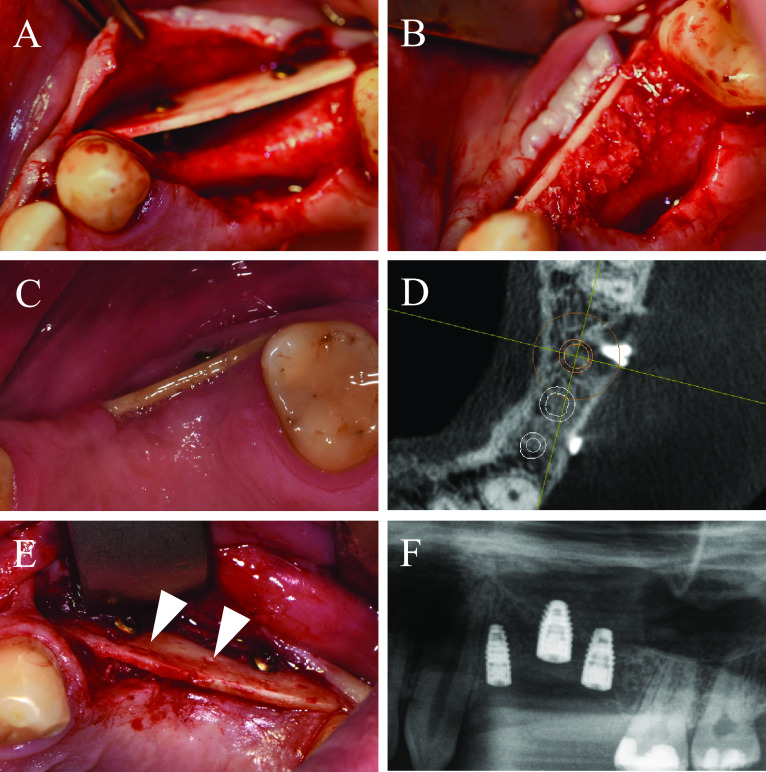
Alveolar ridge augmentation of the maxilla with wound dehiscence. **A** The intraoperative baseline showed an extended horizontal alveolar ridge atrophy. An allogeneic cortical bone plate was fixed laterally to the defect by two adjusting screws. **B** The space between the allogeneic cortical bone plate and the local alveolar ridge bone was filled with spongious allogeneic granules and the area was covered by a porcine pericardial matrix. **C** After a few weeks, a dehiscence defect with an exposure of the allogeneic CP was found. **D** Surgical re-evaluation demonstrated a plate fracture along the screw holes (white arrows). **E** CBCT scan demonstrated a sufficient bone volume for implant placement. **F** X-ray demonstrating the inserted implants

A 25-year-old patient presented himself after multiple tooth loss of the right maxilla and an extended horizontal tub-shaped and partially vertical bone atrophy and a wish for an implant-borne prosthetic restoration. In the past, alveolar ridge augmentation has been performed using autogenous bone block from the mandibular angle with a failure of the transplant after a few weeks because of transplant infection. Therefore, in accordance to patient’s wishes, the current treatment plan included a re-augmentation using CP. After palatinal-shifted crestal incision, a full-thickness flap was raised with marginal alleviations. After preparation of the recipient site by using a sharp raspatorium, the defect size was measured and CP was extra orally prepared, placed and fixed by two adjusting screws (each 1.5 × 10 mm) (Fig. 6A). The space between the CP and the local bone was filled with allogeneic granules and the augmented area was covered by a porcine pericardium collagen membrane (Fig. 6B). After 6 weeks, an asymptomatic and painless dehiscence defect was found (Fig. 6C). Extensive mouth hygiene was performed with mouth rinsing solutions (0.2% chlorhexidine), up to three times a day for 6 weeks. After 3 months, CBCT scan was performed to analyze the current bony situation and to plan implant placement showing sufficient horizontal bone volume (Fig. 6D). After 4 months, the augmented area was revisited. The inserted CP showed a plate fracture alongside the screw line (Fig. 6E). The upper part of the broken CP was removed after removal of the adjusting screws. The local bone was sufficient for implant placement and three implants (BLT, 3.3/4.1 × 10 mm, Straumann) were placed (Fig. 6F). Within the follow-up of 40 months, the case remained stable without complications.

### Space filling

**Fig. 7:**
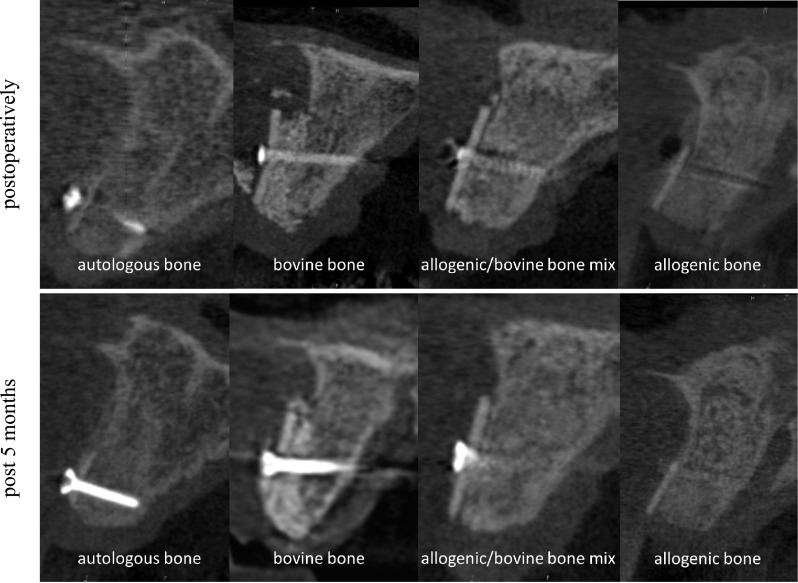
Alveolar ridge augmentation with different fillings, autologous bone, bovine substitute, a mixture of allogeneic bone and bovine substitute, and allogeneic bone at CBCT scans. Upper line situation postoperatively and lower line situation after 5 months in CBCT scans

Filling the space between CP and local bone is comparable with filling defects in the context of guided bone regeneration (GBR). There are differences between the barrier function of CP and the membranes used for GBR as CP has a high-volume stability and is rigidly connected to the alveolar bone. Overall, space between CP and local bone can be filled in different ways. CBCT scans demonstrated the complete remodeling of autogenous and allogeneic granules after 5 months radiologically. On the contrary, bovine BS seems to be integrated, but radiological remodeling seems to be less compared to autogenous and allogeneic granules. On the contrary, the combination of allogeneic and bovine bone granules demonstrates a sufficient integration and partial remodeling in part with inclusion of bovine BS radiologically (Fig. 7). Up to date, detailed studies about the filling materials are missing, especially after loss to the CP. With respect to CBCT scans, use of autogenous and allogeneic granules can be recommended, especially since both groups demonstrate a complete remodeling process [44].

### Histology

**Fig. 8:**
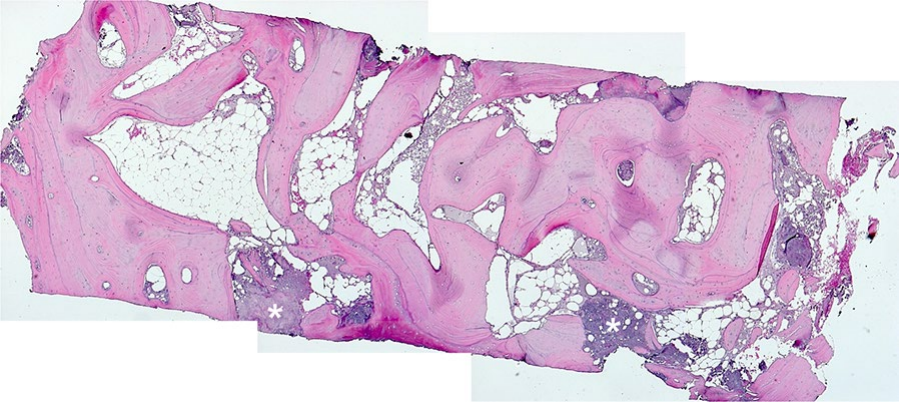
Overview after reconstruction of single sections, newly formed cancellous bone, white asterisks = detritus, HE staining, original magnification × 5

**Fig. 9:**
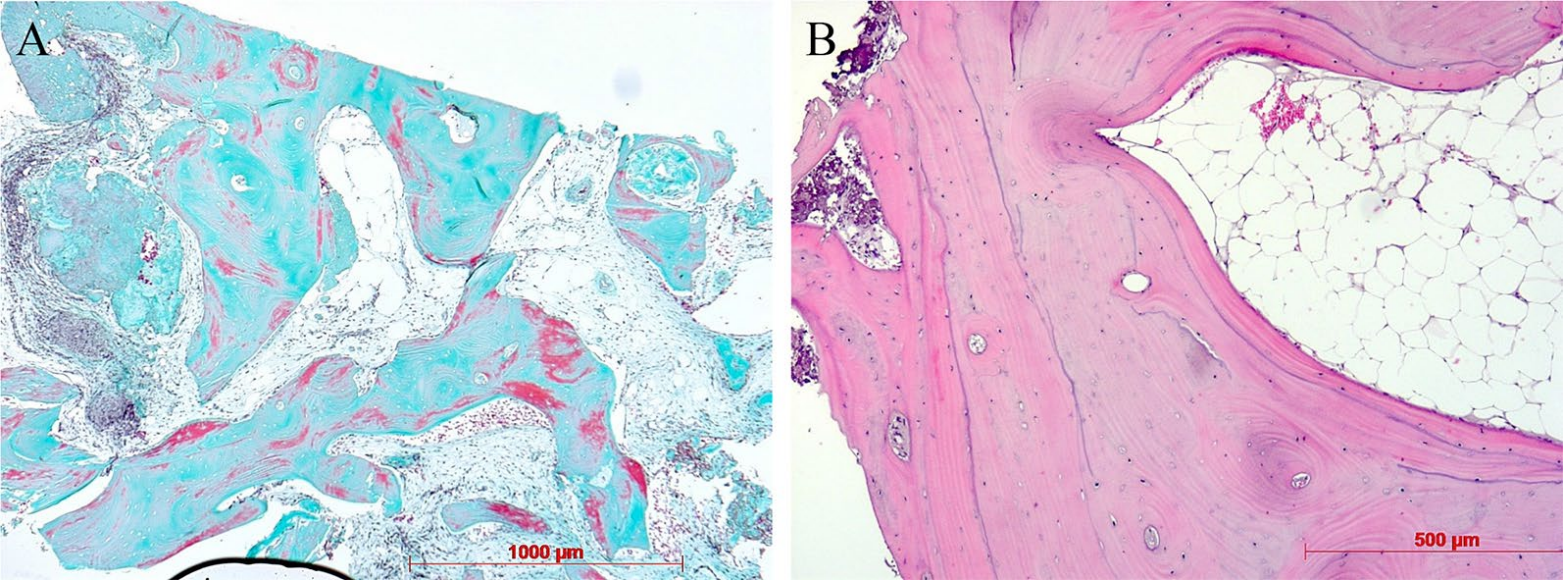
Newly formed cancellous bone, intertrabecular loose connective tissue, trichrome staining, original magnification × 5 **(A)**. Newly formed cancellous lamellar bone, HE staining, original magnification × 10 **(B)**

**Fig. 10:**
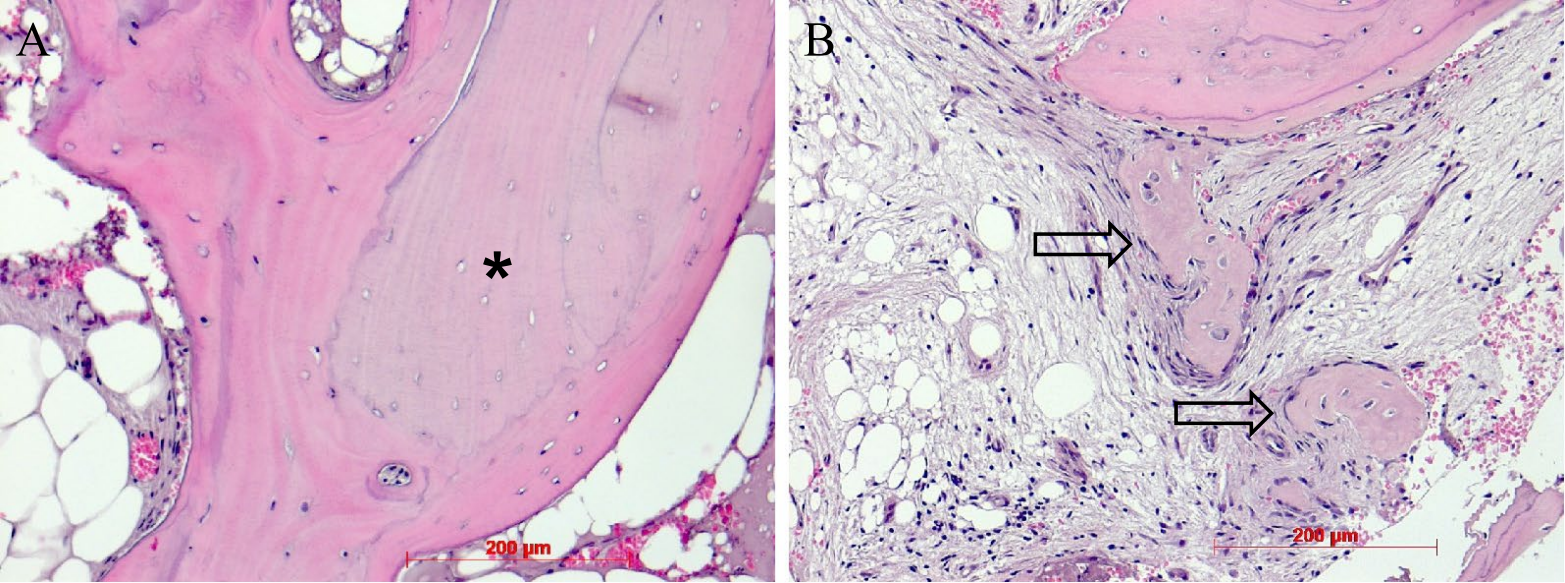
Newly formed cancellous lamellar bone, embedded allogenous remnant (black asterisk), HE staining, original magnification × 20 **(A)**. Ongoing membranaceous osteogenesis, osteoblast covering (arrows), HE staining, original magnification × 20 **(B)**

Artificial ecchymosis and bone or connective tissue fragmentation due to trephination could be observed in all specimens (Fig. 8). All biopsies showed advanced osteogenesis by forming lamellar, mature cancellous or compact bone. Next, the formation of a network of cancellous bony trabeculae with intertrabecular vascularized loose connective tissue could be observed (Figs. 8, 9). Allogeneic particles could be clearly identified as mostly basophilic lamellar bone fragments containing empty osteocyte lacunae embedded into newly formed bone (Fig. 10A). Focally, ongoing membranaceous osteogenesis forming woven bone ossicles covered by osteoblasts was visible (Fig. 10B). A nearly compact lamellar bone was formed (Figs. 8, 9). In two cases, no allogeneic grafts or graft remnants could be found. However, embedded remnants of woven bone indicate former membranaceous osteogenesis followed by remodeling into lamellar bone. In all cases, no osteoclast or very few small osteoclast appearances indicate missing or very slow resorption. No signs of inflammation or necrosis could be observed.

## Discussion

This case series reported about more than 300 cases with different types of alveolar ridge defects that were sufficiently reconstructed by allogeneic shells. Main findings were that this technique is able to facilitate a sufficient bone gain with high-quality bone, an overall low complication rate and a restricted number of implant failures. As a special feature, implant placement was possible in nearly all cases with plate complications with very limited derogation. Most frequent complications were dehiscences that could be considered as common complications in different kinds of alveolar ridge augmentation [45–47]. In this context, dehiscences could be an even more frequent problem or complication in larger or extended augmentations. Sufficient soft tissue management and plate preparation as well as augmentation within the bony envelope might minimize the potential risk of an augmentation site exposure. In this context, a preoperative qualitative and quantitative soft tissue assessment can be highly recommended [48]. Overall, the procedure can be summarized as safe, but it requires extended surgical skills. One of its main advantages is the waiving of autogenous graft harvesting and therefore the absence of a second surgical field for graft harvesting and a risk for donor site morbidity, such as nerve damage. The shell technique with CP can be used in different types of defects, such as trilateral-walled tub-shaped defects and extended and combined vertical and horizontal defects. Next to one single CP, even two or more CP can be placed from different sites to the defect. An over-augmentation outside the bony envelope should be avoided since it might result in an increased bone resorption and dehiscence [49]. After smoothening of sharp edges of the plate via a rotating instrument, the plate was fixed by screws of a length of 8–10 mm and a diameter of 1–1.5 mm. It is important to use straight screws which must not have a conical extension towards the screw head in order to reduce shearing forces to the plate. The augmentation area can be covered by a collagen membrane for stability of the particles which does not seem to be necessary if only autogenous granules cover the top of the augmented area. The most relevant aspects concerning the avoidance of complications are:

1. rehydration of CP by at least 10 min, e.g., in 0.9% saline to prevent plate fracture,
2. trimming and drill hole preparation outside the oral cavity,
3. stable CP positioning and fixation by adjusting screws,
4. strict smoothing of sharp and pointed edges of CP,
5. augmentation within the bony envelope as well as
6. a tension-free wound closure.

Complication management (e.g., dehiscence defects) should include an optimized oral hygiene in combination with mouth rinsing solutions (0.2% chlorhexidine), up to three times a day. It should be avoided to remove the CP in cases without a putrid infection or pain or other compelling reasons.

In contrast to different autogenous bone shell augmentations, a crestal, horizontally placed shell to cover the augmentation area in the vertical dimension was not applied in this case series, since it seems not to be associated with further advantages (e.g., an increased stability of the augmentation area or an improved bone remodeling) according to the authors’ experiences. In contrast, it could be possible that a crestal, horizontally placed CP could cause further complications, such as wound dehiscences. This topic could be addressed in a further clinical study. Based on our experiences, using allogeneic shells seems not to compromise the clinical results and patients’ outcome and can be considered as a suitable alternative to autogenous shells whereby a conclusive comparison is unfeasible. Appreciated benefits of autogenous bone shells are the long-time experience and well-documented, excellent clinical results in combination with low complication rates and an absence of immunologic reactions or transmission of infectious diseases [29]. Next, autogenous bone grafts are distinguished by their osteoinductive characteristics that might be a further advantage especially in compromised patients [50]. Possible limitations are donor site morbidity, such as nerve irritations and injuries, wound healing disturbances, bleeding and pain [28, 29, 51]. Even the mandibular integrity might be influenced after bone harvesting from the jaw angle [52]. In comparison, notable advantages of allogeneic shells are the reduced surgery time since harvesting, extraoral shell preparation and supply of the donor region are missing, the absence of donor site morbidities, and the unlimited graft availability. This latter point might be of special interest in cases where bone from the jaw angle has already been harvested in the course of previous therapies [28]. In addition, the results of this case series demonstrated sufficient clinical, radiological and histological results for allogeneic shells that appear to be similar to those of autogenous shells. Focusing on clinical data, Tunkel et al. compared autogenous and allogeneic shells with autogenous space filling in a split-mouth model and found no differences concerning horizontal and vertical bone gain and resorption rates within the groups. Implant placement could be performed in all cases [28]. A case series reported about 4 patients with alveolar ridge augmentation using CP, allogeneic granules and a porcine pericardium membrane. Re-investigation at implant placement demonstrated a sufficient bone remodeling and vascularization within the three-dimensional CP containers [53]. This can be confirmed by our histological findings. In contrast, Khojasteh et al. retrospectively analyzed the block tenting technique for vertical and horizontal alveolar ridge defects using autogenous vs. allogeneic blocks as shells and BS filling. Highest horizontal bone gain was found for autogenous ramus and allogeneic blocks. The highest rate of graft failure was shown for allogeneic blocks [54]. It remains unclear whether graft failure might be associated with pure BS filling. Therefore, this could be an important clue concerning the relevance of different space filling materials or mixtures. A study analyzed possible differences between allogeneic spongious and cortico-spongious granules and a mixture of allogeneic/autogenous granules for alveolar ridge augmentation. After 18–20 weeks, histological analysis revealed new bone formations of about 25, 29 and 26%, respectively, without significant differences within the groups [55]. Similar results were demonstrated for ridge preservation [56]. With respect to the current study, even pure autogenous space filling resulted in sufficient bone formation suitable for sufficient implant placement. Nevertheless, a combination of autogenous granules and bovine BS space filling might be of special interest not least because of it has been reported to be a sufficient mixture for vertical bone augmentation [57]. Next, clinical data showed an adequate clinical and radiological horizontal bone gain in combination with high-quality bone using allogeneic shells and bovine BS filling [58]. The suitability of a bovine BS mixture is further supported by this study demonstrating a sufficient integration and partial remodeling of an allogeneic/bovine BS mixture in part with inclusion of bovine BS radiologically. A mixture using bovine BS might further prevent the need for relining using bovine BS after implant placement to prevent bone resorption and should be further investigated [28]. Next, it has not yet been finally clarified, whether implant survival and success rates (SurR, SucR) in allogeneic grafts are equal to autogenous grafts [2, 59]. Motamedian et al. reported SurR/SucR of 74–100% and 73–100% analyzing 2647 implants in 872 patients in autogenous grafts. Next, SurR/SucR of 95–100% and 94–100% analyzing 1395 implants in 532 patients with allogeneic grafts were found [59]. Unfortunately, to the best of our knowledge, little literature is dealing with information about SurR of implants placed in autogenous or allogeneic shell augmented sites. Simonpieri et al. presented sufficient results in socket preservation with pure allogeneic granules that might be comparable to shell technique with limitations, with implant survival rates of 98% and peri-implant bone loss of less than 1 mm after 4 years follow-up [60]. Another study analyzed the use of allogeneic granules (MBA) in combination with autogenous cortico-spongious plates from the jaw angle. Four to six months after alveolar ridge augmentation, implants were placed to the augmented areas. In total, 1 of 42 inserted implants failed. There was no difference whether the implants were placed in the allogeneic or the autogenous grafts [61]. Overall, there is some indication that implant SurR in allogeneic augmented sites might be comparable to those in autogenous augmented sites. This hypothesis is even reflected in the limited number of implant failures presented in this case series. With a special focus on shell technique, the space filling material seems to be more relevant for implant SurR than the shell material itself. Therefore, further studies should specifically address space filling materials and mixtures with a special focus on implant SurR and SucR. As a limitation, retrospective analysis without a control groups was performed exclusively in this case series. Further randomized controlled clinical trials are required to analyze the topic more precisely.

## Conclusions

To the best of our knowledge, this is the first multicenter case series dealing with data about a large amount of alveolar ridge augmentations using processed allogeneic CP and the shell technique. In conclusion, it can be assessed as a promising alternative to autogenous shells. A final and evidence-based valuation of processed allogeneic CP is not yet possible up to date since the available literature is inhomogeneous with a relevant lack of evidence [2]. Advantages and limitations of autogenous and allogeneic shells have to be carefully considered individually in each case to determine the best solution for the patient. A following study could address implant survival and success rates, even with a special focus on different filling materials.

## Data Availability

Data and material will be available upon a reasoned request.
